# European Society of Cardiology congress update, Rome, 27–31 August 2016

**Published:** 2016

## Introduction

The annual European Society of Cardiology (ESC) meeting was held at the Nuova Fiera di Roma with over 32 000 delegates from 126 countries in attendance.

The meeting commenced with an outstanding address on the heart and art by a British cardiac surgeon, who demonstrated the amazing discoveries in cardiac anatomy and function made by Leonardo da Vinci over 500 years ago, and the awarding of the ESC gold medal to Dr Bernard Gersh of the Mayo Clinic, whose foundational training in cardiology took place at Groote Schuur Hospital.

Four new ESC guidelines addressing atrial fibrillation (AF), heart failure, cardiovascular (CV) disease prevention and dyslipidaemia, as well as a position paper on cardiooncology, were released during the meeting. The full texts ofthese documents are available to all at https://www.escardio.org/ Guidelines/Clinical-Practice-Guidelines.

The meeting planners placed particular emphasis on the ‘heart team’ approach and included a large number of ‘heart hub’ presentations. The latter were delivered ‘in the round’ and provided a more informal, more easily accessible presentation format, which improved interaction between presenters and the audience.

The following are my impressions of the presentations I attended over the five days of the meeting.

## Dyslipidaemia

The 2016 dyslipidaemia guideline has been harmonised with the CV disease prevention guideline, which appeared simultaneously. The ESC has maintained the SCORE risk factor charts as well as a chart estimating relative risk in younger people. The risk categories have likewise been maintained. However, whereas the presence of significant plaque on carotid ultrasound classifies the patient as very high risk, increased carotid intima–media thickness does not. Treatment targets have been maintained. Very high-risk patients have a low-density lipoprotein cholesterol (LDL-C) target of < 1.8 mmol/l, high-risk subjects < 2.6 mmol/l and moderate- to low-risk individuals < 3.0 mmol/l. In patients with diabetes an HbA1c < 7% is recommended in addition. In very high- and high-risk patients, treatment should achieve a > 50% reduction in LDL-C. High-density lipoprotein cholesterol (HDL-C), apoB/apoA1 and non-HDL-C/HDL-C ratios are not recommended as treatment targets. Statins remain first-line treatment, given up to the highest recommended dose or highest tolerable dose to achieve the treatment goal. Statin treatment should be given for the same indications and using the same targets in women and the elderly.

A small Japanese study from the Heart Institute of Japan involving 1 734 patients with dyslipidaemia followed for 3.9 years after acute coronary syndrome (ACS) found no benefit from the addition of ezetimibe to pitivastatin vs pitivastatin alone. LDL-C was 1.7 mmol/l in the combination group vs 2.2 mmol/l in the statin-only group.

Patients with heterozygous familial hypercholesterolaemia (FH) respond inadequately to statin therapy and frequently require plasma apheresis to lower their LDL-C. Apheresis is both expensive and inconvenient for the patient. A study evaluating the PCSK9 inhibitor, alirocumab, demonstrated a 75% reduction in the need for apheresis.^1^ Unfortunately many patients were not taking statins during the study, so the effect of the combination of PCSK9 inhibition, statin therapy and apheresis could not be determined (Table 1).

**Table 1:** ESC CP guidelines 2016: dyslipidaemia

*Treatment targets*
*2011 ESC dyslipidaemia guidelines*			*2016 ESC dyslipidaemia guidelines*		
*Recommendation*	*Class*	*Level*	*Recommendation*	*Class*	*Level*
Very-high CV risk: LDL-C goal < 70 mg/dl (1.8 mmol/l) and/or 50% reduction when target cannot be reached	I	A	Very-high CV risk: LDL-C goal < 70 mg/dl (1.8 mmol/l) and/or 50% reduction if baseline is 70–135 mg/dl (1.8–3.5 mmol/l)	I	B
High CV risk: LDL-C goal < 100 mg/l (2.5 mmol/l)	IIa	A	High CV risk: LDL-C goal < 100 mg/l (2.6 mmol/l) or 50% reduction if baseline is 100–200 mg/dl (2.6–5.1 mmol/l)	IIa	B
Moderate CV risk: LDL-C goal < 115 mg/l (3.0 mmol/l)	IIa	C	Moderate CV risk:LDL-C goal < 115 mg/l (3.0 mmol/l)	IIa	B

## Hypertension

A session on hypertension dealt with the problems of masked and white-coat hypertension (WCH). Masked hypertension is defined as a normal office blood pressure, but elevated home blood pressure or 24-hour ambulatory blood pressure readings. Home monitoring (generally recorded at rest) and ambulatory blood pressure (recorded over 24 hours) measure different aspects of the blood pressure profile. Masked-hypertension patients are, by definition, untreated. Masked uncontrolled hypertension (MUCH) is seen in treated hypertensives. The blood pressure typically fluctuates and elevations may occur either during waking hours or at night (typically in obstructive sleep apnoea). Masked hypertension is present in 10–20% of the population and doubles the risk of a CV event.

There is currently no guidance from clinical trials as to the correct treatment of masked hypertension. The MASTERS trial commenced recently to explore what the correct treatment should be. At present the recommendation is to establish strict risk factor control and, though ‘logically’ incorrect, not to institute antihypertensive therapy.

WCH affects 30–40% of the hypertensive population. Patients with WCH have a metabolic profile similar to that of hypertensive patients. Their baseline blood pressure is marginally higher than that of the normotensive population. The WCH group has a higher incidence of increased pulse pressure and left ventricular hypertrophy and a higher 10-year risk of developing established hypertension and diabetes. The prognosis of WCH is intermediate between normotensive and hypertensive patients with a higher incidence of CV events. There are no therapeutic strategies proven to be effective in WCH.

**Fig. 1.**
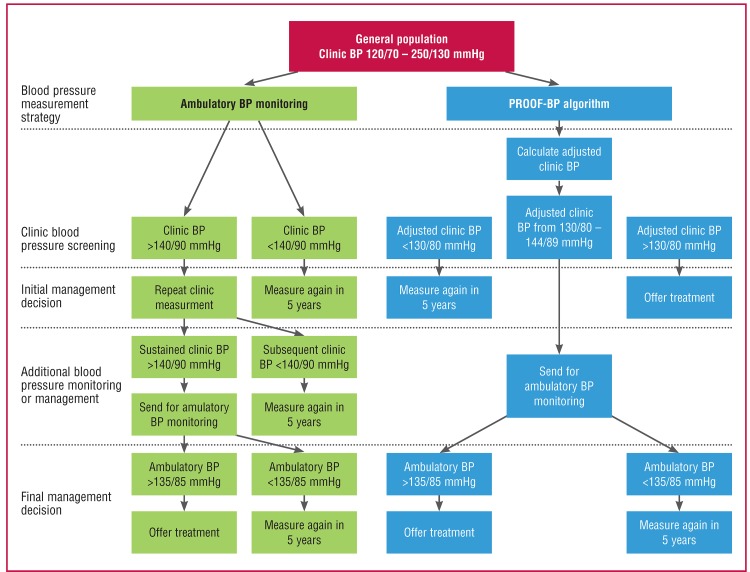
Algorithm for using the Predicting Out-of-Office Blood Pressure in clinic tool (PROOF-BP) prediction model to triage patients for out-of-office blood pressure monitoring. Existing strategies are based on the hypertension diagnostic pathway specified by the US Preventive Services Task Force and the National Institute for Health and Care Excellence. BP indicates blood pressure. Source: Hypertension 2016; 67(5): 941–950.

McManus and Sheppard evaluated the application of multiple serial office blood pressure readings to reasonably predict out-of-hospital blood pressure levels. Their data from the PROOF blood pressure study support the value of obtaining ambulatory blood pressure recordings in patients with an office blood pressure of 130/80 to 144/89 mmHg, therefore requiring ambulatory blood pressure recordings in 58% of patients.^2^ They have published an algorithm for calculating home blood pressure (https://sentry.phc.ox.ac.uk/proof-bp) (Fig. 1).

Kario from Japan pointed out that central aortic pressure is a further component to be considered in blood pressure control. It is best controlled with an angiotensin converting enzyme inhibitor (ACE-I) or angiotensin receptor blocker (ARB) and not with beta-blockade. Diurnal variation in blood pressure and aortic stiffness are other aspects receiving attention. He commented that when combined with an ARB (olmesartan), hydrochlorothiazide is superior to calcium channel blockade for the control of nocturnal hypertension.

A lunchtime symposium was devoted to the underuse of mineralocorticoid antagonists (MRAs), namely spironolactone and eplerenone, in both hypertension and heart failure. Both have proven benefits in improving blood pressure control in resistant hypertension^3^ and patient survival in heart failure. Hypertension is controlled best at a serum potassium level of around 4.5 mmol/l, but concerns about the development of hyperkalaemia result in avoiding appropriate prescription or under-dosing of MRAs. The survival benefit for heart failure patients is not eliminated in those who develop hyperkalaemia. However, the detection of a high potassium level most often leads to the discontinuation of the whole spectrum of renin–angiotensin–aldosterone system (RAAS) inhibitors and a consequent loss of efficacy. Hyperkalaemia may be controlled in the short term by calcium carbonate, alkalinisation, beta-stimulants, insulin and potassium, and Na^+^ polystyrene sulphonate (Kayexalate). Patiromer^4^ and Na^+^ zirconium cyclosilicate^5^ are two agents currently in development that control hyperkalaemia in the long term and may enable the uninterrupted continuation of therapeutic doses of RAAS inhibitors.

The CLARIFY study analysed data from 22 672 patients with stable coronary artery disease (CAD) and treated hypertension. It found an increase in cardiovascular events in those with systolic blood pressure > 140 mmHg and diastolic blood pressure > 80 mmHg. However systolic blood pressure < 120 mmHg and diastolic blood pressure < 70 mmHg were also associated with an increase in cardiovascular events, except for stroke. This finding supports the presence of a J-shaped curve in relation to outcomes and the blood pressure achieved on treatment.^6^

## Prevention

The 2016 guidelines on the prevention of CV disease target smoking, diet, physical activity, body weight, blood pressure, serum lipids and diabetes. Management should be individualised after assessment of personal risk using the SCORE tables. No additional predictive accuracy derives from the measurement of biomarkers. In apparently healthy individuals, risk assessment should be carried out from the age of 40 years in men and 50 years in women or when they are post-menopausal. Risk assessment may be repeated at five-year intervals in those with no identifiable risk factors and more often in those with risk close to threshold. Broadly speaking, management targets are avoidance of any exposure to smoking, a diet low in saturated fat, activity for at least 150 minutes each week, a body mass index (BMI) of 20–25 kg/m^2^, blood pressure < 140/90 mmHg, LDL-C according to the guideline on dyslipidaemia (i.e. < 3.0 mmol/l in low- to moderate-risk individuals) and, in patients with diabetes, an HbA_1c_ level < 7%. Aside from the general recommendations, the guidelines contain specific recommendations for the management of patients with hypertension, CAD, chronic heart failure, cerebrovascular disease and peripheral vascular disease.

A ‘naturally randomised’ study evaluated outcomes dependent on whether the study population’s LDL-C and blood pressure lay above or below the median, therefore evaluating lifelong exposure to these recognised risk factors. The differences between the lower and higher groups were LDL-C 0.31 mmol/l and blood pressure 5 mmHg. Outcomes were better in those with a lower LDL-C or blood pressure with a multiplicative effect observed when both were below the median.

Population data have been collected in Finland since 1972. At the outset, the regions in the east of the country had the world’s highest incidence of CAD. A programme to encourage behavioural change was launched, including, smoking cessation and a reduction in dairy product intake. Since then there has been a continuous decline in CV disease of 4.4% per annum, thus achieving a cumulative reduction of 80% since the start of the project. Two-thirds of the decline is ascribed to risk factor management and one-third to medication and intervention.^7^

The Europe-wide survey EURO-ASPIRE IV demonstrates persistent overweight and obesity in the population. Eightyeight per cent of respondents were found to be overweight with two-thirds exhibiting central obesity. Women were more frequently affected than men.

A study from the Netherlands examined medication compliance in 59 000 patients after either ST-elevation myocardial infarction (STEMI) or non-ST-elevation myocardial infarction (NSTEMI). It showed that only 34% of patients took all five of the recommended therapies. STEMI patients were more likely to adhere (43%) than those with NSTEMI (28%). The latter group was more likely not to be taking statins and antiplatelet therapy. A Korean study showed that medication adherence can be improved by simplifying the timing of daily administration.

The PALM registry found that underdosing of secondary preventative therapies was prevalent and that untreated and underdosed patients exhibited a higher LDL-C value.

The OPTICARE study involved an educational and interventional programme in patients after ACS, comparing standard care versus a face-to-face physical training programme combined with counselling, versus a programme of telephone contact. More than 80% of patients completed the programme. A high percentage received optimal medical therapy (OMT). The study found no benefit to patients from more intensive management.

Depression and anxiety are frequent concomitants in patients with CAD (25%) or stroke (33%). Its occurrence is more frequent with advancing age, elevated blood pressure and alcohol use. In the study reported at the meeting, effective secondary preventative treatment did not influence the rate of depression or anxiety. Very few patients had received treatment for their depression/anxiety.

Bisphosphonates may reduce arterial calcification and so influence the progression of atherosclerotic disease. A retrospective analysis showed that the cardiovascular mortality rate was reduced by 19% and all-cause mortality by 10%.

An international randomised study in 2 717 patients with prior CAD or stroke, who had obstructive sleep apnoea, showed that the use of continuous positive airway pressure (CPAP) over four years failed to improve cardiovascular outcomes. Quality of life was improved. The apparatus was used on average only for around 3.5 hours each night. Fewer stroke events were noted in those who used CPAP for more than four hours per night.^8^

## Coronary artery disease

Several reports were predicated on concerns about the high incidence of normal coronary angiograms in patients investigated for suspected stable CAD. Most quoted an incidence of 60–70% without obstructive disease. The CONSERVE trial over 12 months used coronary computed tomography angiography (CCTA) first, to assess whether invasive angiography was required. This approach reduced the assessed need for invasive angiography by 78%, revascularisation from 17 to 10% and the cost by 50%. In the Clinical Evaluation of Magnetic Resonance Imaging in Coronary Heart Disease 2 trial (CE-MARC 2), the NICE guidelines were compared to cardiac magnetic resonance (CMR) and to myocardial perfusion imaging. This reduced the ‘unnecessary’ angiography rates to 28, 7 and 7%, respectively. In both studies, the MACE rates were not impacted on by avoidance of angiography.^9^

The PACIFIC study carried out a head-to-head comparison of CCTA, myocardial perfusion single-photon emission computed tomography (SPECT), positron emission tomography (PET) and hybrid imaging in the diagnosis of ischaemic heart disease. The finding was that PET scanning was superior to both CCTA and SPECT.

Plasma apheresis was used in patients with uncontrolled angina receiving maximally tolerated medical therapy. Apheresis improved myocardial perfusion and increased exercise tolerance, as assessed by the six-minute walk test.

The 15-year follow up of the FRISC-II study was reported; it had compared an early invasive strategy in NSTEMI to an initial non-invasive strategy.^10^ Patients who participated are now 80 years old on average. The overall mortality rate has been 40%. Sixty per cent of patients initially treated without intervention have subsequently undergone revascularisation. The frequency of unplanned revascularisation followed a parallel trajectory in the two groups after three to four years. CV death or MI was ‘postponed’ by three to four years in the intervention group, which also experienced a substantial reduction in the frequency of rehospitalisation. These benefits were seen in those patients who were troponin positive at the time of enrollment. By contrast, the 10-year follow up of the ICTUS study again found no benefit from early intervention, with the incidence of MI driven by peri-procedural events.

A Japanese trial, which had compared ad hoc to deferred PCI in patients with stable CAD, reported its five-year outcomes. There were no differences in incidence of death or MI. Deferred cases fared better when heart failure was present, but the deferred group also had a higher incidence of stroke.

Widimsky presented a small trial (1 230 patients) from the Czech Republic that looked at one-month outcomes in STEMI patients who received either prasugrel or ticagrelor (PRAGUE-18 study). No differences were discernible at seven and 30 days. Due to financial constraints, many patients had to switch to clopidogrel after discharge, frustrating the assessment of effect at a later time point.^11^

NORSTENT included 9 013 patients receiving their first coronary stent [either bare-metal (BMS) or newer drug-eluting stents (DES)] between 2008 and 2011.^12^ Seventy-one per cent of cases were treated for ACS. Eighty-four per cent of procedures were performed by the radial route. Forty per cent of patients had multi-vessel disease. An average of 1.7 stents was implanted per patient. Dual antiplatelet therapy (DAPT) was given for nine months in both groups.

Median follow up was for five years. There were no differences in outcome between the two types of stent. Repeat revascularisation was 3.3% less with DES. Stent thrombosis occurred in 0.8% with DES and 1.2% in BMS patients. Quality of life was no different between the two groups.

In the LEADERS FREE trial, patients over 75 years of age at high risk of bleeding received either a BioFreedom® polymerfree drug-coated stent or Gazelle® BMS and DAPT for only one month. Their average age was 81 years, 63% had multi-vessel disease and one-third had atrial fibrillation (AF). Sixty per cent of procedures were performed via the radial artery. At one year the event rate was 14 versus 11% in favour of the BioFreedom® stent, the difference being driven by the incidence of MI. There was a 49% reduction in target-vessel revascularisation and no increase in rate of bleeding.^13^

A 10-year follow up of the SIRTAX trial showed an increase in non-CV mortality rate between five and 10 years and a constant rate of MI, but a fall-off in the incidence of target lesion revascularisation and stent thrombosis with no difference between paclitaxel- and sirolimus-eluting stents. The use of DAPT, aspirin and statin treatment was observed to be declining.^14^

A five-year follow up of the trial comparing Biolimus® (biodegradable polymer) to sirolimus-eluting stents showed some crossover of benefit in favour of Biolimus® with regard to rates of death, MI, stent thrombosis and target-vessel revascularisation.^15^

A two-year follow up of the ABSORB® stent (bio-absorbable vascular scaffold) study found four instances of very late stent thrombosis. Optical coherence tomography showed that undersizing of the stent and discontinuities in stent structure might have been the cause.^16^

The DOCTORS study compared optical coherence tomographyguided to angiography-guided intervention in localised single-vessel disease. Fractional flow reserve (FFR) results were moderately improved when using optical coherence tomography.

The BBK II trial compared TAP stenting with Culotte stenting in bifurcation lesions, demonstrating that the Culotte technique yielded better results in the side branch. Commentators cautioned against use of the Culotte technique by those who are not experts.^17^

Jang, from Harvard Medical School, reported on an optical coherence tomography-guided study in Chinese patients with ACS, which identified plaque erosion as the underlying cause in 30%. These patients were treated with aspirin and ticagrelor without stenting. He showed that thrombus volume was diminished at one month.^18^

A sub-study of the DAPT trial evaluated whether OMT (using ACE-I/ARB, beta-blockade, statin, thienopyridine and aspirin when indicated by guidelines) influenced the outcome of prolonging DAPT. The benefit of DAPT was shown to be consistent, whether or not patients were receiving OMT.^19^

The ITALIC trial two-year result showed no difference between six months and 24 months of DAPT (predominantly using clopidogrel). However, there was a trend towards increased events in patients with prior MI who received only six months of DAPT.

A study of an ‘as-treated’ subgroup of the FREEDOM trial compared coronary artery bypass grafting (CABG) to percutaneous coronary intervention (PCI) in patients with diabetes and multi-vessel CAD, both with and without chronic kidney disease. CABG was associated with a lower incidence of death and MI but with an increase in the risk of stroke.^20^

The STITCH trial evaluated the benefit of CABG in patients with left ventricular systolic dysfunction (LVSD). CABG benefited patients in all age groups. CV mortality was the most frequent cause of death at all ages. Non-cardiac causes of death were more frequent in the elderly.

BASKET-SAVAGE compared BMS to DES in saphenous vein graft stenting with the option of using a filter wire and/ or GPIIb/IIIa inhibitors. DES were associated with an 80% reduction in MACE (12 vs 30%) driven by non-fatal MI and target-vessel revascularisation. There was no difference in mortality rate. The contribution of the filter wire and GPIIb/IIIa inhibition used in 67% of cases could not be determined.

## Heart failure

The 2016 heart failure guideline discusses means to prevent or delay the onset of symptomatic heart failure. Predominant among these are treatment of hypertension, statins for those with CAD or at high risk thereof, smoking cessation, avoidance of excess alcohol intake, an ACE-I for patients with asymptomatic LVSD, and beta-blockade for those with LVSD after MI.

Heart failure is defined as appropriate symptoms, possibly accompanied by physical signs of congestion, with evidence of structural heart disease on echocardiography. The guideline includes a new category of heart failure with mid-range ejection fraction (HFmrEF) of 40–49% in the range between heart failure with reduced ejection fraction (HFrEF) and heart failure with preserved ejection fraction (HFpEF). Treatment of HFmrEF is yet to be clearly defined.

ProBNP is more important in ruling out heart failure than in proving the diagnosis. Novel additions to the guidelines are the use in appropriately selected patients of an angiotensin receptor neprilysin inhibitor (ARNI) (sacubitril-valsartan) to replace ACE-I, cardiac resynchronisation therapy (CRT), and ivabradine should they remain symptomatic on ACE-I/ARB, beta-blockade and MRA treatment. HFpEF and HFmrEF should be treated symptomatically, using diuretics to relieve congestive symptoms. The guideline discusses at length various co-morbidities and their management in the setting of heart failure.

**Fig. 2.**
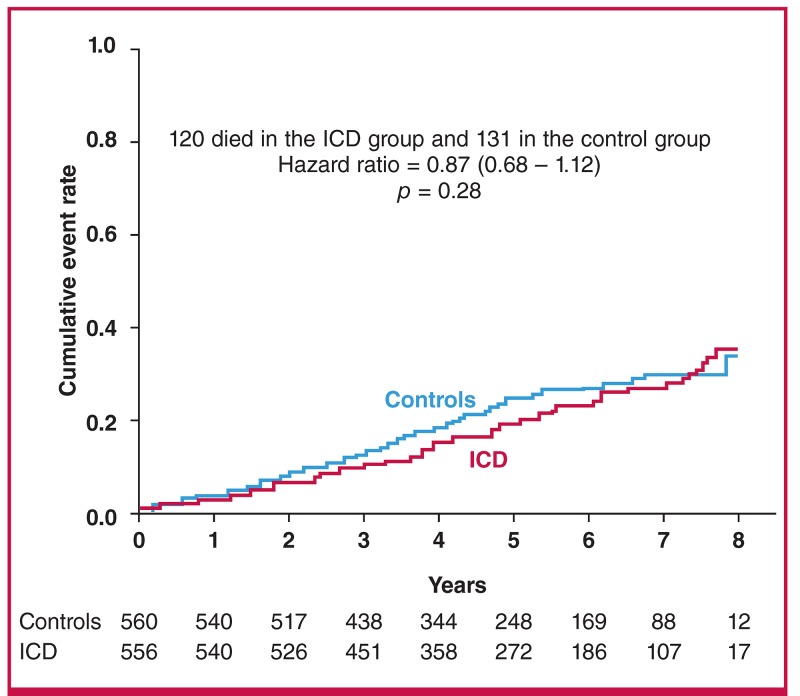
Primary outcome – all-cause mortality

A ‘hotline’ session was devoted to device interventions in heart failure. The DANISH study evaluated the use of implantable cardioverter defibrillators (ICDs) in non-ischaemic heart failure in patients with NYHA class II to III symptoms and an ejection fraction < 35% (Fig. 2).^21^ The study did not achieve its primary endpoint of reducing mortality rates. There was a reduction in incidence of sudden death and a reduction in total mortality rate in the subgroup under 60 years of age. Two other studies, REM-HF and MORE-CARE, reported on remote monitoring for worsening heart failure. Both failed to show any improvement in outcome.

Another two studies on cell therapy in heart failure could not demonstrate any benefit.

## Cardio-oncology

This is an emerging area of concern for cardiologists and oncologists alike, given the increasing numbers of patients who may now survive for years after cancer treatment. The heart and blood vessels may be affected in a variety of ways by either chemotherapy or radiation. Myocardial dysfunction and heart failure, CAD, valvular heart disease, pericardial involvement, arrhythmias, hypertension, pulmonary thromboembolism, stroke, peripheral vascular disease or pulmonary hypertension may occur, not only acutely but also after a delay of months or years.

The patient’s presentation is influenced by the agent used, the dose and duration of treatment, age, kidney function and pre-existing CV disease. Myocardial toxicity is of particular concern and its early detection, as evidenced by deteriorating LVSD on echocardiography in comparison to baseline values and/or by elevation in hs-troponin T, is important. Baseline echocardiography with follow up at the completion of treatment and then at three and six months is recommended. ACE-I or ARB, beta-blocker and MRA therapies are cardioprotective as well as effective in managing overt heart failure. Not all patients recover normal left ventricular function after the cessation of treatment. In those who do, it may be possible to discontinue the cardioprotective treatment.^22^

## Atrial fibrillation

Apart from death and stroke, the ESC 2016 guideline includes recognition of hospitalisations, left ventricular dysfunction and heart failure, cognitive decline and vascular dementia, and impaired quality of life as consequences of AF. An algorithm is provided for the detection of AF in patients with an implanted device presenting after detection of a high atrial rate episode lasting longer than five to six seconds or a rate > 180 beats/min.

For the assessment of stroke risk and the need for anticoagulation, the CHADS-VaSC score remains unchanged but the stroke risk has been reclassified for women. Anticoagulation is indicated in men with a score of 2 or more; for women it’s a score of 3 or more. Anticoagulation is not mandated but may be considered in men and women with respective scores of 1 or 2.

Bleeding risk scores should be considered to determine the presence of modifiable risk factors for major bleeding in patients taking anticoagulant therapy. A non-vitamin K antagonist (VKA)/novel oral anticoagulant is preferred to warfarin. Occlusion of the left atrial appendage may be considered in patients who have a long-term contra-indication to anticoagulation. The guideline provides recommendations for the initiation of anticoagulation after transient ischaemic attack or stroke and the re-initiation of anticoagulation after intracranial bleeding.

The integrated management of AF includes symptom control, maintenance of haemodynamic stability and the preservation of LV function, stroke prevention and the management of CV risk factors. Recommendations for ventricular rate control include consideration of digoxin as a second-line therapy.

ENSURE-AF^23^ examined the use of the factor Xa inhibitor (edoxaban) versus enoxaparin/warfarin-VKA in electrical cardioversion of non-valvular AF in 2 199 patients with and without transoesophageal echocardiography (TEE). The average CHADS-VaSC score was 2.6. The procedure was undertaken with a delay of two and 23 days, respectively, depending on whether or not patients had undergone TEE. There was a very low event rate following cardioversion with a 50–60% reduction in events with edoxaban and no increase in bleeding.

Connolly reported on the ongoing trial of the antidote, andexanet alfa, in patients treated with a factor Xa inhibitor presenting with critical bleeding. Haemostasis was achieved in 79% of patients. However, thrombotic events have been observed after reversal. The 30-day mortality rate was 15%.^24^

## Arrhythmias

The channelopathies include long-QT syndrome (QT interval > 480 ms or > 460 ms in association with syncope and in the absence of factors prolonging the QT interval), short-QT syndrome (QT interval < 340 ms or < 360 ms in the presence of additional features), Brugada syndrome, catecholaminergic polymorphic ventricular tachycardia, early repolarisation syndrome, progressive conduction system disease and idiopathic ventricular fibrillation. Although genetic testing is helpful in a variety of these conditions, it cannot rule out the presence of a particular condition. However once a genetic marker has been identified in a patient, there is a class I indication for testing family members. ‘Overlap’ syndromes may occur.

Depending on the specific diagnosis, the management armamentarium includes lifestyle changes, reduction in the risk of exposure to triggers (e.g. exercise, sudden fright) betablockade (possibly nadolol to be preferred), late sodium channel blockers (e.g. flecainide, propafenone), quinidine, ablation of an ectopic focus and consideration of an ICD or pacemaker. Fever and alcohol exposure should be avoided in Brugada syndrome.

Priori presented her basic science research on gene therapy in mice using adeno-associated virus (AAV) infection to either add an active gene or silence a mutant. Problems may be posed by the high incidence of antibodies to this virus.^25^

## Valvular heart disease

In a session on mitral valve repair, Obadia illustrated several surgical approaches to the mitral valve. Alifieri discussed the problems associated with mitral valve repair. He emphasised the importance of recognising that ‘everything is closer than you think’: the commissure between the left and non-coronary aortic valve leaflets, the circumflex coronary artery, the artery to the atrioventricular node, the atrioventricular node itself and the coronary sinus all lie in close proximity to the mitral annulus and risk being injured during repair. It is challenging to remove calcification from the mitral annulus. As a result, mitral leaks in the region of the posterior annulus are seen more frequently in the elderly. Mitraclip®, discussed by Latib, may be complicated by inadequate grasping of the leaflet or leaflet perforation. Occasionally systolic anterior motion of the mitral anterior leaflet may result in intermittent mitral regurgitation during exercise only; this may require provocation with isoprenaline to demonstrate its presence.

It is important to recognise that in patients with aortic regurgitation, only 50% have primary aortic valve disease; the primary pathology is in the aorta in the other half. Sinotubular dilatation, aortic dissection and occasionally aortic dissection flap prolapse may be at fault. Three-dimensional echocardiography is to be preferred over two-dimensional to quantify the degree of aortic regurgitation. A left ventricular end-systolic diameter > 50 mm or left ventricular end-diastolic diameter > 70 mm predicts the need for surgery. However, earlier referral for surgery is recommended in symptomatic patients and those with LVSD. TEE is strongly recommended prior to referral for surgery as well as intra-operatively.

The feasibility of aortic valve repair depends upon the pliability of the leaflets and their freedom from calcification. Repair of a bicuspid aortic valve is less successful, especially when decalcification and cusp repair with a pericardial patch is required. There are many smaller studies reporting successful aortic root repair with freedom from re-operation and absence of residual regurgitation.

## Venous thromboembolism

Computed tomographic pulmonary angiography (CTPA) is frequently used in suspected acute pulmonary embolism (PE). The majority of these costly investigations yield a negative result. The YEARS project devised a simplified algorithm for diagnosing acute PE. The first step is to obtain a D-dimer test and score 1 point for each of the following: clinical assessment for signs of deep venous thrombosis (DVT), haemoptysis and whether PE is the most likely diagnosis. If the YEARS score is 1 or more or if the D-dimer value is < 1 000 ng/ml, order CTPA. If not, PE can be ruled out and CTPA is unnecessary. This method has been shown to be safe and will reduce the frequency of CTPA by 14% overall and to a greater degree in younger patients.

In DVT-PE the outcome is not influenced by whether treatment is initiated with rivaroxaban or with enoxaparin with later bridging to rivaroxaban.

Following treatment for venous thromboembolism (VTE), it is problematic to decide whether anticoagulant therapy may be safely withdrawn owing to the potential for recurrent events. Rodger presented a validation of the ‘men continue and HERDOO2 rule’ which identifies those at low risk of recurrence in whom anticoagulation can be withdrawn. The rule states that all males and certain females scoring 2 or more using the HERDOO rule must continue anticoagulation long term after unprovoked or minor provoked VTE. The HERDOO factors are HER: hyperpigmentation, oedema or redness in either leg, D: level of D:dimer assessed through blood testing, O: obesity defined as BMI ≥ 30 km/m^2^, and O: older than 65 years of age.
